# Digital interventions for healthy ageing and cognitive health in older adults: a systematic review of mixed method studies and meta-analysis

**DOI:** 10.1186/s12877-023-04617-3

**Published:** 2024-03-04

**Authors:** Yvette I-Pei Tsai, Jeanie Beh, Charlotte Ganderton, Adrian Pranata

**Affiliations:** 1 School of Nursing & Midwifery, University of Newcastle, Callaghan, Australia; 2 Centre for Design Innovation, Swinburne University of Technology, Melbourne, Australia; 3 School of Health and Biomedical Sciences, RMIT University, Melbourne, Australia

**Keywords:** Ageing, Technology, Health knowledge, Dementia risk, Digital engagement

## Abstract

**Background:**

Currently, there is no systematic review to investigate the effectiveness of digital interventions for healthy ageing and cognitive health of older adults. This study aimed to conduct a systematic review to evaluate the effectiveness of digital intervention studies for facilitating healthy ageing and cognitive health and further identify the considerations of its application to older adults.

**Methods:**

A systematic review and meta-analysis of literature were conducted across CINAHL, Medline, ProQuest, Cochrane, Scopus, and PubMed databases following the PRISMA guideline. All included studies were appraised using the Mixed Methods Appraisal Tool Checklist by independent reviewers. Meta-analyses were performed using JBI SUMARI software to compare quantitative studies. Thematic analyses were used for qualitative studies and synthesised into the emerging themes.

**Results:**

Thirteen studies were included. Quantitative results showed no statistically significant pooled effect between health knowledge and healthy behaviour (I^2^ =76, *p=*0.436, 95% CI [-0.32,0.74]), and between cardiovascular-related health risks and care dependency I^2^=0, *p=*0.426, 95% CI [0.90,1.29]). However, a statistically significant cognitive function preservation was found in older adults who had long-term use of laptop/cellphone devices and had engaged in the computer-based physical activity program (I^2^=0, *p<*0.001, 95% CI [0.01, 0.21]). Qualitative themes for the considerations of digital application to older adults were digital engagement, communication, independence, human connection, privacy, and cost.

**Conclusions:**

Digital interventions used in older adults to facilitate healthy ageing were not always effective. Health knowledge improvement does not necessarily result in health risk reduction in that knowledge translation is key. Factors influencing knowledge translation (i.e., digital engagement, human coaching etc) were identified to determine the intervention effects. However, using digital devices appeared beneficial to maintain older adults’ cognitive functions in the longer term. Therefore, the review findings suggest that the expanded meaning of a person-centred concept (i.e., from social, environmental, and healthcare system aspects) should be pursued in future practice. Privacy and cost concerns of technologies need ongoing scrutiny from policy bodies. Future research looking into the respective health benefits can provide more understanding of the current digital intervention applied to older adults.

**Study registration:**

PROSPERO record ID: CRD42023400707 https://www.crd.york.ac.uk/prospero/display_record.php?RecordID=400707.

**Supplementary Information:**

The online version contains supplementary material available at 10.1186/s12877-023-04617-3.

## Background

The number of people aged over 60 years is increasing worldwide [46]. Consequently, there is an increasing number of age-related diseases such as cardiovascular disease, depression, chronic pain, dementia, and cognitive decline [9, 46]. In Australia, healthcare costs for age-related diseases, specifically dementia-related care, are estimated to be over 3 billion of the total healthcare expenditure [2]. These costs are predicted to grow by 3.33% every year [12].

With the population ageing at an accelerated rate, healthy ageing has become a global healthcare agenda [44]. The main characteristic of healthy ageing is considered a person’s intrinsic mental and physical capacity, within their environment (e.g., social interaction), to function in everyday life [46]. To age successfully, a person’s health is defined not only by disease absence but also by optimising and maintaining the quality of everyday life [38].

Dementia is not an automatic consequence of ageing. However, dementia has a substantial relation to age-related diseases and causes significant disability and dependency among the older population [45]. As a neurocognitive disorder, dementia currently has no cure and there is limited evidence-based intervention proven effective in preventing the onset of dementia [24]. However, many health risks including obesity, physical inactivity, and unhealthy diet, are considered modifiable to mitigate age-related diseases. Thus, targeting age-related health risks to promote healthy ageing is seen as a preventative measure to reduce dementia risk development in the ageing population [46].

Digital technologies for older adults, anecdotally termed gerontechnology, have been utilised in many aspects of healthcare. They may appear in telehealth used in primary care or smartphone applications used to support mental (i.e., cognitive training) and physical health (i.e., exercise programs) [39]. However, currently, there is no systematic review investigating the effects of each available digital intervention applied to the older population. Therefore, this review aims to answer the following research questions:

1. How effective are digital interventions to facilitate healthy ageing and cognitive health of older adults?
2. What are the considerations of digital interventions to support healthy ageing and cognitive health for older adults?

### Review design and methods

This review was conducted using a systematic review approach and guided by the Joanna Briggs Institute mixed-method systematic reviews [25]. The review was reported in accordance with the Preferred Reporting Items for Systematic Reviews and Meta-Analyses (PRISMA) [32]. The review of quantitative studies allowed the study to evaluate the effectiveness of the digital interventions. Whereas the review of qualitative studies provides further understanding of the considerations influencing the digital intervention effects. The review protocol was registered with PROSPERO (CRD42023400707).

### Search strategy

Six databases were searched including CINAHL, Medline, ProQuest, Cochrane, Scopus, and PubMed. The main search terms were digital health, older people, and dementia. While there were limited results after three terms altogether, two terms were interchangeably searched, e.g., digital health AND older people, or digital health AND dementia. Detailed search terms are included in Supplementary Appendix 1.

### Inclusion and exclusion criteria

This review included all types of intervention studies and used the Population, Intervention, Comparison, Outcome (PICO) framework to determine the eligibility of the study inclusion or exclusion.

#### Types of studies

This review included quantitative and qualitative studies that conducted an intervention using digital technologies to facilitate healthy ageing and maintain cognitive health of older adults including reducing the risk of cognitive decline or dementia in older adults. Randomised Controlled Trials (RCT) were included. Non-randomised controlled Trial studies included quasi-experimental, cohort, or quantitative components in the mixed method study. Qualitative studies included descriptive, explanatory, or ethnographic studies.

#### Population

This review included older adults with a mean age of greater than or equal to 55 years. Study populations primarily with dementia or cognitive impairment were excluded. However, studies were included if their intervention was primarily on healthy older adults but also included participants with mild cognitive impairment or dementia. This allowed the review to examine the intervention effects on slowing cognitive decline or reducing dementia risk for the purpose of maintaining/sustaining the cognitive health of older adults. This review focused on the up-to-date evidence-based data source. Only journal research articles that were published in the last 10 years and published in English were included.

#### Intervention

Studies that conducted an intervention using digital technology in older adults were included. Digital technology in this review was defined as any tool, device or resource that contains an electronic digital format. Studies that did not involve digital technology in the intervention or evaluation of the digital intervention were excluded.

#### Comparison

Studies using comparison or control groups in the intervention were included. The digital interventions without a comparison group were also included.

#### Outcome measure

The primary outcome of this review was the effect of digital intervention on promoting healthy ageing in older adults. Healthy ageing was considered in various areas relating to physical and mental health addressed in the study intervention for older adults. The secondary outcome was the effect of the digital intervention on maintaining the cognitive health of older adults. This included interventions aimed at slowing cognitive decline and reducing the risk of dementia to maintain the cognitive health of older adults. Cognitive functions were measured by the study using cognitive assessments such as the Mini-Mental State Examination (MMSE), Montreal Cognitive Assessment (MoCA), Clinical Dementia Rating Scale (CDR), global cognition z-score, Repeatable Battery for the Assessment of Neuropsychological Status (RBANS) test, and Cardiovascular risk factors, Ageing and Incidence of Dementia (CAIDE). Digital interventions were grouped into categories based on the types of technology they used. The effects of the quantitative intervention outcomes were measured by statistical significance via the study-reported p-values. The effects of the qualitative intervention outcomes were measured by the study themes or the study-reported evaluation of user feedback.

### Study selection and data extraction

To structure the study selection and data extraction process, the Preferred, Reporting Items of Systematic Reviews and Meta-Analyses (PRISMA) were followed [29]. Data synthesis was completed using Covidence systematic review software, Veritas Health Innovation, Melbourne, Australia [10]. Titles and abstracts obtained from the search strategy were screened by two independent reviewers (YT and AP). Any disagreement on the study inclusion or exclusion was further assessed by the third reviewer (CG). All authors (YT, AP, JB, CG) independently reviewed the full-text articles based on the eligibility criteria and completed data extraction.

### Quality assessment

Quality assessment of included studies was assessed using the Mixed Methods Appraisal Tool Checklist (MMAT) [16]. The MMAT is a critical appraisal tool that allows the assessment of five study categories including qualitative, randomised control trials, non-randomised trials, and quantitative descriptive and mixed method studies. Each category set out five criteria for assessing the methodological quality and converting the assessment results into a score between 0 (low quality, high risk of bias) and 5 (high quality, low risk of bias). Each reviewer assessed the study quality independently and met to discuss the quality scores. Any discrepancy in the scores was further assessed by the third reviewer. A further level of evidence matrix using Stichler’s [41] method was applied to appraise the hierarchical quality of evidence with each study MMAT result. This level of evidence matrix allowed the review to weigh each study from level 1 indicating highly reliable evidence to level 6 indicating the least reliable evidence.

### Data synthesis

The convergent integrated approach was applied to synthesise quantitative, qualitative and mixed-method studies [25]. The process occurred concurrently to combine extracted data from the studies.

#### Quantitative data

Wherever possible, quantitative studies with homogenous data were grouped to analyse their outcome measures reported in dichotomous or continuous data to synthesise the intervention effect for meta-analysis. The meta-analysis was done with the inverse variance analysis method and presented in forest plots as odd ratios for dichotomous data and standard mean differences for continuous data in JBI SUMARI software [31]. Heterogeneity between the studies was assessed by using I-squared (I^2^) tests where an I^2^ statistic value larger than 50% was considered substantial [14]. The overall effect of the studies was assessed by p-value where p ≤ 0.05 indicates statistically significant. Sensitivity analyses were performed by using a repeated measure to test the meta-analysis results. Where a meta-analysis was not possible, the quantitative data were synthesised with narrative descriptions.

#### Qualitative data

The data analysis was carried out using thematic analysis [8]. A mixture of inductive and deductive approaches was employed during the analysis process. Firstly, a complete reading of the results and conclusions was carried out with the different included studies. Secondly, information corresponding to the research questions of this review was identified, using the authors’ interpretations and textual quotes. The textual descriptions were then extracted directly from each qualitative study and assembled into several codes. Finally, main themes and sub-themes emerged and led to the main findings of this review. The entire process was developed by two reviewers (YT and JB) where the coding was initially done by one reviewer (JB) and checked by another reviewer (YT). The codes were then grouped and synthesised into emerging themes by one reviewer (YT) and reviewed by a third independent reviewer (AP).

## Results

### Study selection

**Fig. 1:**
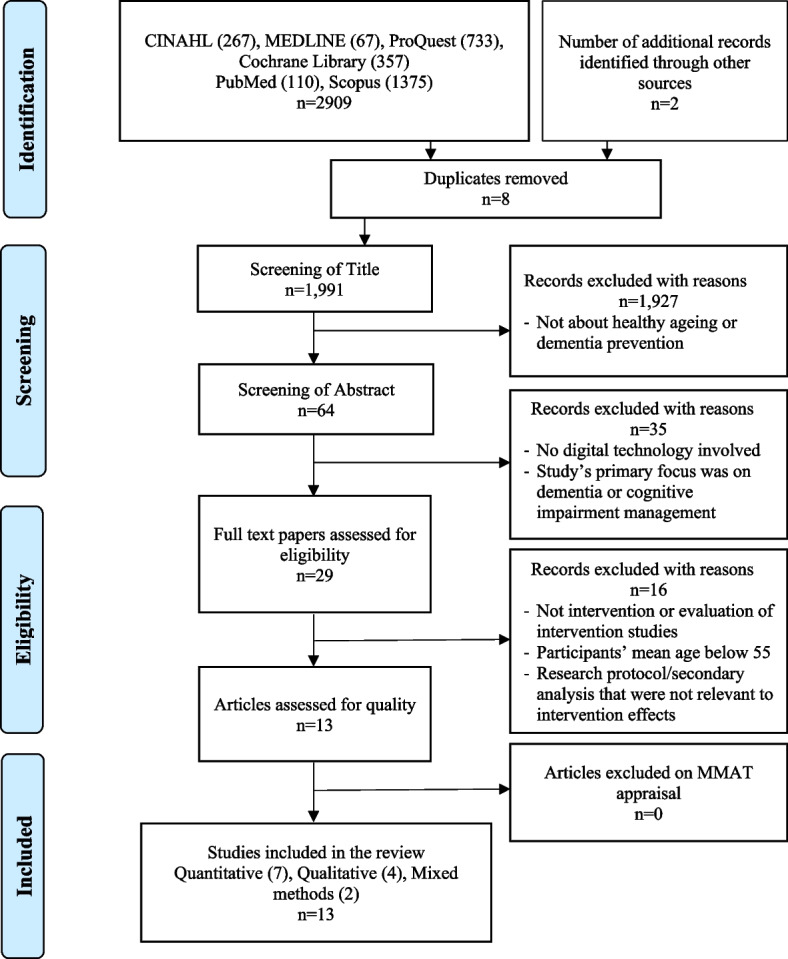
Preferred Reporting Item for Systematic Reviews and Meta-Analyses (PRISMA) flow chart for literature search [30]

The database search yielded 2,909 articles. After applying limiters and removing duplicates, 1,991 articles were screened for title and abstract, and 29 were included for full-text screening. The eligibility of one study [43] appeared to be dissent between the reviewers due to the lack of a specific participant group outcome. Hence, the corresponding author of the article was contacted for further information. The study was included during the selection process, and the disagreement was resolved by all authors reaching a consensus on the quality assessment of the study. Sixteen studies were excluded from the 29 full-text screening. Of the 16 studies excluded, two studies were excluded because one was the phase one result of a research protocol, and another was the primary outcome from the study’s secondary analysis. The phase one results of a research protocol have the same results published in the research article that had been included in the review. The primary study of the secondary analysis was excluded because it was not related to the intervention or evaluation of the intervention. Another two studies were excluded because participants’ mean age was below 55. The rest of the twelve studies were excluded because they were not related to intervention or evaluation of intervention research. The final 13 studies were included in this review. Figure 1 summarises the study selection process adhering to the PRISMA guideline.

### Study characteristics and quality

Table 1 summarises the included study characteristics. Of thirteen studies included in this review (n_total_=19,551participants). Seven studies were quantitative research [6, 13, 17, 19, 22, 35, 43] (*n=*19,245 participants). Four studies were qualitative research, [3, 4, 18, 33] (*n=*260 participants). Two studies were mixed methods research [27, 42] (*n=*73 participants). Participants’ mean age ranged between 58 and 80 years. Three studies [3, 22, 43] (*n=*177) included both cognitively intact participants and participants with mild cognitive impairment, dementia, and cognitive decline. Only three studies specifically focused on improving the cognitive health of older adults [19, 22, 43] (*n=*13,651 participants), whereas two study interventions [17, 35] (*n=*2,871 participants) aimed to improve healthy ageing and cognitive health. For the meta-analysis of quantitative data, four quantitative studies presented dichotomous data [13, 19, 35, 43] and three quantitative studies presented continuous data [6, 17, 22].

**Table 1:** Study characteristic summary

**Study** **Location/technology type**	**Purpose**	**Population (n)**	**Study method**	**Intervention**	**Outcomes**	**MMAT**	**Level of evidence**
Richard et al [35] Location: Netherlands, Finland, and France Type: Assistive/information technology	To investigate whether a coach-supported interactive internet intervention to optimise self-management of cardiovascular risk factors in older individuals can improve cardiovascular risk profiles and reduce the risk of cardiovascular disease and dementia	People aged ≥ 65 years or over at increased risk of cardiovascular (CV) disease *n=*2724 (f:1297, m:1427) *n=*1389, IG *n=*1335, CG	Quantitative: Longitudinal, randomised-controlled trial	An interactive internet intervention stimulating coach-supported self-management or a control platform that involves guided goal setting, monitoring, personalised coaching, lifestyle group activities, information on cardiovascular health and risk factors.	Data available for 2398 (88%) participants. IG compared to CG showed: - Increased composite score of systolic BP, LDL, BMI, *p=*0.008 - Decreased systolic BP (mean difference: -1·12 mmHg) - Decreased BMI (mean difference: –0·15 kg/m^2^) - Decreased LDL level (mean difference: -0·05 mmol/L)	Score: 5 Category 2 – Yes – Yes – Yes – Yes 2.5 – Yes	Level 2
Jin et al. [19] Location: China Type: Assistive technology	To examine the independent protective factors of desktop and cell phone ownership, or combined ownership, against cognitive decline in mid-life and older adulthood	Age 45 and over (mean 58) *n=*13,457 (f:6867, m: 6590) Desktop: *n=*2314 Control: *n=*11143 Cell phone: *n=*10693 Control: *n=*2764	Quantitative: Longitudinal cohort study	Ownership of a computer with internet connection, and cell phone.	- Participants with a desktop had less cognitive decline over the four years, *p=*0.003 - Participants with a cell phone had less cognitive decline, *p<*0.001	Score: 5 Category 3 3.1 – Yes 3.2 – Yes 3.3 – Yes 3.4 – Yes 3.5 – Yes	Level 2
Vicentin et al. [43] Location: Brazil Type: Information technology	To evaluate the effectiveness of combined digital inclusion and physical activity interventions in the prevention of cognitive and functional loss among elderly residents	Older adults with normal to mild cognitive impairment >60 years *n=*112 (f:86, m:21) *n=*53, IG *n=*54, CG	Quantitative: Comparative controlled study	Computer-based digital inclusion program combined with physical activity 80-minute sessions twice a week for 17 weeks)	- IG showed a significantly higher MoCA mean score after 4 months by 1.23 points, *p=*0.012 than the CG - No significant differences after 4 months for MMSE, GDS, Word List, Evocation, Verbal Fluency, and ADL in the IG when compared to CG	Score: 4 Category 3 3.1 – Yes 3.2 – Yes 3.3 – Can’t tell 3.4 – Yes 3.5 – Yes	Level 2
Hsu et al. [17] Location: Taiwan Type: Information technology	To implement and evaluate a cross-disciplinary health education intervention program using two approaches in community-based older adults for the purpose of successful ageing	Older adults aged > 70 years old *n=*147 (f:114, m:33) Intervention group: *n=*61 (person-to-person), *n=*54 (person-to-digital) Control group: *n=*32	Quantitative: Quasi-experimental, multi-centre design	Lecture-based person-to-person (P2P) and person-and-digital (P&D) education program in community care centres for 12 weeks 9-components: concept and preparation for healthy ageing, PA, nutrition and diet, chronic disease prevention and management, emotional health and coping skills, cognitive function training, family relationship, financial security, and internet use	- P&D group had a significant reduction in nutrition risk, *p<*0.05 - Cognitive function increased over time for all groups, *p* <0.01 - Both P2P and P&D groups significantly increased in the selection adaptation strategy, *p* <0.01 - P2P group had a significant effect on the use of emotion-focused coping, *p* <0.05 - P&D group significantly increased its ability to search for health information online, *p* <0.05	Score: 3 Category 3 3.1 – Yes 3.2 – Yes 3.3 – No 3.4 – Yes 3.5 – Can’t tell	Level 2
Hasemann et al. [13] Location: Germany Type: Information/communication technology	To investigate the effectiveness of a multi-component community-based healthcare approach for functional impairments in the elderly	Age ≥ 70 *n=*2,670 (f:1752, m:918) *n=*873 IG *n=*1,797 CG	Quantitative: Quasi-experimental study	Multi-component care approach that involved: - geriatric screening - case management - community-based activities of prevention and health promotion - digital supporting tools (e.g., tablet, online platform)	- No significant difference in the progression of long-term care grade between groups, *p=*0.616 - No intervention effects for long-term care grade, mortality, and health-related quality of life - Statistically significant relative change in morbidity, *p=*0.006 for the intervention group.	Score: 4 Category 3 3.1 – Yes 3.2 – Yes 3.3 – Yes 3.4 – Yes 3.5 – No	Level 2
Kumar et al [22] Location: United States Type: Information technology	To evaluate the impact of a remotely delivered multidomain lifestyle intervention, the virtual cognitive health (VC Health) program, on the cognitive function and mental health of older adults with subjective cognitive decline	Older adults aged 60-74 years old with subjective cognitive decline scoring ≥1 on the Subjective Cognitive Decline Questionnaire (SCD-9) *n=*82 (f:61, m:21)	Quantitative: Prospective, single-arm, intention-to-treat, pre-post, remote nationwide clinical trial	Virtual Cognitive Health 12-month Program components: individually tailored coaching sessions on nutrition, physical exercise, and cognitive training (including processing speed, executive function, working memory, episodic memory, and mental speed)	Cognitive measures tested: - Mean increase of 5.8 in RBANS Total Index score from baseline to week 52, *p<*0.001 - Mean decrease of 3.8 units in PHQ-9 score from baseline to week 52, *p<*0.001 - Mean decrease of 2.9 units in GAD-7 survey score from baseline to week 52, *p<*0.001	Score: 3 Category 3 3.1 – Yes 3.2 – Yes 3.3 – No 3.4 – can’t tell 3.5 – Yes	Level 2
Bevilacqua et al. [6] Location: Italy Type: Information technology	To evaluate an innovative eHealth five-part training module focused on enhancing digital learning opportunities, literacy, skill acquisition usage, and fostering a culture of later-life learning.	Older adults aged over 50 years old *n =* 58 (f:24, m:34)	Quantitative: Observational cohort study	Five modules over a 4-week training program using the GoToMeeting platform	- eHealth literacy value improved significantly from baseline to follow-up, *p=*0.001 - significant relationship between eHealth literacy and survey of technology use, *p=*0.032 - significant relationship between satisfaction with training and eHealth literacy, *p=*0.000 - 22.8% of the users would pay for the course, *p=*0.004	Score: 3 Category 4 4.1 – Can’t tell 4.2 – Can’t tell 4.3 – Yes 4.4 – Yes 4.5 – Yes	Level 3
Ienca et al. [18] Location: Switzerland Type: Assistive/communication technology	To explore views, needs and perceptions of community-dwelling older adults regarding the use of digital health technologies for healthy ageing	Cognitively healthy community-dwelling older adults aged > 65 years *n =* 19 (f:9, m:10)	Qualitative	Four digital health systems: A toy-shaped conversational robot; a smartphone application for care coordination; two wrist-worn wearable devices	Main themes: - General value of digital assistive technologies - Usability evaluations - Ethical considerations	Score: 5 Category 1 1.1 – Yes 1.2 – Yes 1.3 – Yes 1.4 – Yes 1.5 – Yes	Level 3
Pettersson et al. [33] Location: Sweden Type: Information technology	To explore older people’s experiences of a self-management falls prevention exercise routine guided either by a digital program (web-based or mobile) or a paper booklet	Community-dwelling participants ≤70 years with self-reported impaired balance *n =* 67 (f:19, m:9)	Qualitative	Self-managed exercise program involving 10 self-paced exercises delivered digitally via video or using a paper booklet.	Main themes: - Participants expressed both a capability and willingness to independently manage their exercise. - A digital program strengthens the feeling of support while creating their own exercise program and tailoring it to their preferences and circumstances Subthemes: - Finding my own level - Programming it into my life - Evolving my acquired knowledge - Defining my source of motivation	Score: 5 Category 1 1.1 – Yes 1.2 – Yes 1.3 – Yes 1.4 – Yes 1.5 – Yes	Level 3
Baldassar et al. [4] Location: Australia Type: Communication technology	To investigate the importance of distant support networks and the role of new communication technologies for the support and well-being of older Australians from migrant and non-migrant backgrounds	Older migrants aged over 55 *n=* 150 older adults from 10 countries	Qualitative: ethnographic research	Digital communication technologies (e.g., phone, video calls, social media platforms)	Main themes: - Digital kinning practices support the access of older migrants to: - Essential sources of social connection and support - Maintenance of cultural identity - Protection of social identity, including across distance. - Effectiveness of digital kinning is reliant on access to affordable and reliable digital communication tools	Score: 5 Category 1 1.1 – Yes 1.2 – Yes 1.3 – Yes 1.4 – Yes 1.5 – Yes	Level 3
Balasubramanian et al. [3] Location: United Kingdom Type: Assistive technology	To explore the user experience of a compact tablet device to support ordinary people’s everyday living and potential impact on their health and well-being in real-world settings	Older adults aged 50-90 with diagnosed medical conditions *n =* 44 patients *n =* 7 informal carers *n =* 27 focus group	Qualitative	A smart speaker with voice control was installed in participants’ homes. This device includes a screen and speaker with voice control that relays personal digital assistance with various built-in skills that have a wide range of applications.	Main themes: - Self-management and autonomy - Impact on the lifestyle habits - Impact on the mental and social well-being	Score: 5 Category 1 1.1 – Yes 1.2 – Yes 1.3 – Yes 1.4 – Yes 1.5 – Yes	Level 3
Mair et al. [27] Location: Singapore Type: Assistive technology	To describe the development, feasibility, effectiveness, and acceptability of a personalised smartphone-delivered just-in-time adaptive intervention (JITAI) to support older adults to increase or maintain their PA level in a free-living setting	Older adults aged 56-72 years *n =* 46 (f:17, m:14)	Mixed Methods	A wearable activity tracker (Fitbit) and a companion smartphone app (JitaBug) that delivered goal setting, planning, reminders, and just-in-time adaptive intervention messages to encourage achievement of personalized PA goals.	- 67% completed the intervention. - On average, participants recorded 50% of the voice memos, 38% of the mood assessments, and 50% of the well-being assessments through the app - Acceptability of the intervention was very good (77% satisfaction) - Participants suggested a need for more diverse and tailored PA messages, app use reminders, technical refinements, and an improved user interface	Score: 5 Category 5 5.1 – Yes 5.2 – Yes 5.3 – Yes 5.4 – Yes 5.5 – Yes	Level 3
Sungur et al. [42] Location: Netherlands Type: Communication technology	To evaluate a web-based oncological module that integrates with a Health Communicator app to stimulate healthcare participation and improve satisfaction among older Turkish-Dutch and Moroccan-Dutch patients with Cancer.	27 Turkish-Dutch and Moroccan-Dutch older patients with cancer aged 50 years and older and cancer survivors *n=*27 (f:18, m:9) *n=*15 Turkish *n=*12 Moroccan *n=*12 Health care professionals (GPs and oncology nurses	Mixed Methods	Individual survey of question prompt lists (QPL) before and after health professional consultation Patients watched videos via smartphones Phone interviews after the video watch	- A strong correlation between the ease of using the QPL and patient age, harder for older patients to use QPLs, *p=*0.01 - Younger age reported more convenience in using QPL before consultation, *p=*0.003 - Health professionals rated QPL as useful and easy to use - Patients most asked questions were treatment-related information - Overall, patients reported being highly satisfied with their consultations - Overall, patients found the tool useful in improving their communication with the healthcare professionals	Score: 4 Category 5 5.1 – Yes 5.2 – Yes 5.3 – Yes 5.4 – Yes 5.5 - No	Level 3

*IG* intervention group, *CG* Control Group, *PA* physical activity, *RBANS* Repeatable Battery for the Assessment of Neuropsychological Status, *PHQ-9* Patient Health Questionnaire9, *BP* blood pressure, *BMI* body mass index, *MMSE* mini-mental state examination, *GDS* global dementia scale, *ADL* activity daily living, *GAD-7* Generalized Anxiety Disorder-7, *GP* general practitioner

The study appraisal using the MMAT is detailed in Table 1. Study quality assessments from the MMAT scores were between 3 and 5 indicating moderate to high study quality, with a low to moderate risk of bias. Table 2 shows the level of evidence matrix with MMAT score. The evidence matrix of each included study falls between levels 2 and 3, indicating a moderate to high level of study evidence [41].

**Table 2:**
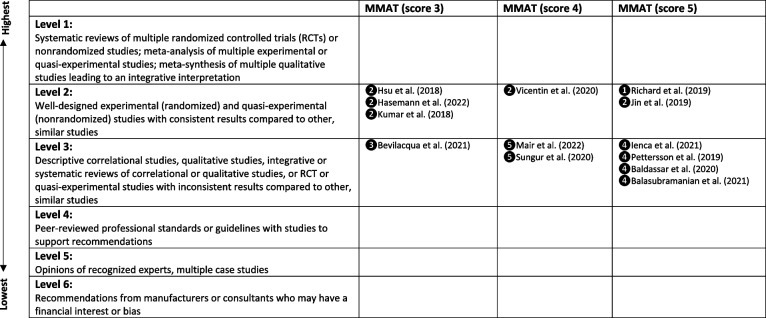
Level of evidence matrix with MMAT score [4, 3, 6, 13, 17–19, 22, 27, 33, 35, 42, 43]

**❶**RCT

**❷**non-randomized

**❸**Quantitative descriptive

**❹**Qualitative

**❺**Mixed methods

Stichler, J. F. [41]. Weighing the Evidence.

*HERD Health Environments Research & Design Journal*,* 3*(4), 3-7 https://doi.org/10.1177/193758671000300401

All studies used digital technology to facilitate healthy ageing or maintain the cognition of older adults. Ten studies focused on healthy ageing in various health areas, including health literacy, self-health management, physical activity, social isolation, care dependency, health service communication, and assistive home living. Three studies focused on maintaining the cognitive health of older adults, including sustaining cognitive function by utilising technology to slow further cognitive decline or reduce the risk for dementia [19, 22, 43]. Two studies addressed their interventions for both healthy ageing and cognitive health of older adults [17, 35].

### Type of digital intervention

**Fig. 2:**
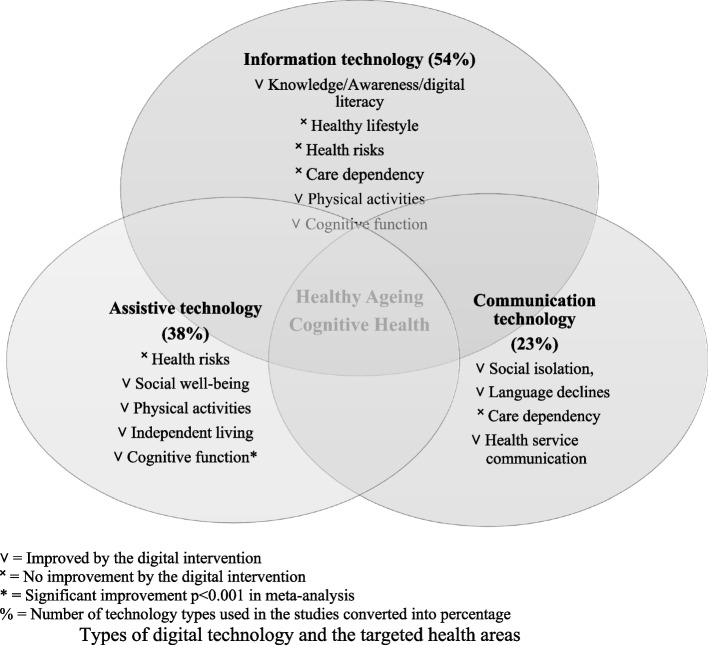
Types of digital technology and the targeted health areas. **˅** = Improved by the digital intervention. **˟** = No improvement by the digital intervention. * = Significant improvement *p<*0.001 in meta-analysis . % = Number of technology types used in the studies converted into percentage

The commonly used digital technology for older adults in the reviewed studies were information, assistive and communication types of technology. Seven studies implemented information type of technology (i.e., website program, digital learning platform) to deliver educational content influencing older adult’s knowledge, awareness, lifestyle, physical activities, and cognition [6, 13, 17, 22, 33, 35, 43]. Five studies incorporated an assistive type of technology (i.e., computer, mobile application, smart home device) to support the well-being of people with health conditions, reduce health risks and physical inactivity of older adults and observe the impact of the technology on persons’ cognitive function over time [3, 18, 19, 27, 35]. Three studies utilised communication technology (i.e., video calls and social media platforms) to reduce social isolation, language decline of older migrants, care dependency and health service communication [4, 13, 42]. Three studies appeared to include hybrid-type technology, including both assistive and communication types [18], communication and information types [13] or assistive and information types [35]. Figure 2 summarises types of digital technology and the targeted health areas.

### Effectiveness of digital intervention for healthy ageing and cognitive health

Studies that use digital technology to facilitate healthy ageing in older adults can be summarised into the improved health knowledge and increased physical activities but had no change in health risk reduction and care independence. Studies that use digital technology for cognitive health found it to maintain the cognitive function of older adults when using digital devices (e.g., laptops or cellphones) or engaging in computer-based physical activities in the longer term. There were also improved dementia risk scores from cardiovascular risk reduction and improved depression, anxiety and the associated risks for dementia from the digital programs. The following sections synthesise the review findings from the quantitative and mixed-method studies and meta-analysis.

#### Health knowledge for healthy behaviour

**Fig. 3:**
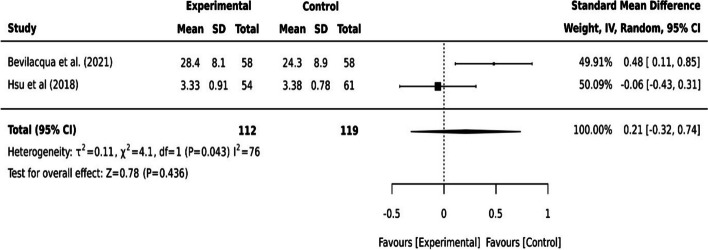
Continuous data for health knowledge to successful ageing

Online training programs and digital learning platforms were utilised to promote health knowledge, healthy behaviour, digital literacy and competency [6, 17]. Compared to the conventional method of content delivery (face-to-face), older adults in the digital format group had increased ability in health information search (*p<*0.01), knowledge of nutrition status (*p<*0.05) and adaptation to ageing (*p<*0.05) [17]. Digital health literacy examined by the eHealth literacy scale in Bevilacqua et al. [6] also showed a statistically significant improvement in participants’ health knowledge after the digital training program (*p=*0.001). However, the overall satisfaction with Bevilacqua et al. [6] online training program was not statistically significant (*p=*0.107). The increased knowledge to health behaviour and mental health were not statistically significant in Hsu et al. [17] digital program. The pooled effect of these two digital programs [6, 17]on health knowledge to healthy behaviour showed not statistically significant (I^2^ =76, *p=*0.436, 95% CI [-0.32,0.74]) (see Fig. 3).

#### Physical activities and health risk reduction

**Fig. 4:**
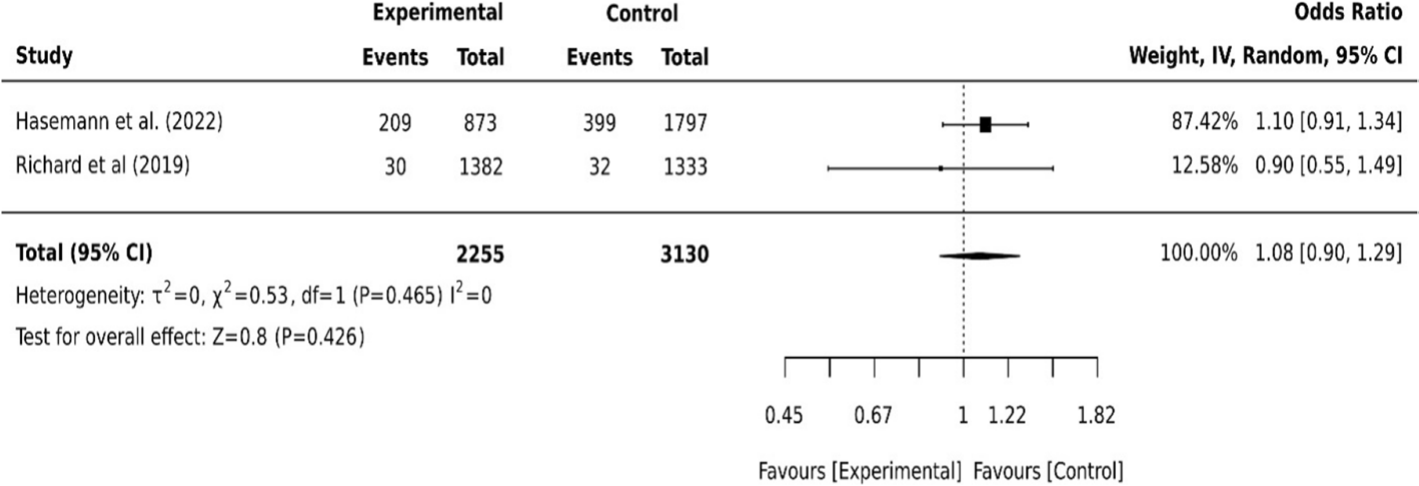
Dichotomous data for cardiovascular risks and care dependency

Digital devices were incorporated into online training programs to increase older adults’ physical activity and reduce cardiovascular-related health risks and the risk of care dependency [13, 17, 27, 35]. A wearable tracker with a smartphone application increased older adults’ engagement in their daily physical activities [27]. However, the digital education program to improve regular exercise by Hsu et al. [17] did not show statistically significant (*p=*0.084). For health risk reduction, older adults in the coach-supported internet platforms had no statistically significant effect on cardiovascular risk (*p=*0.10) and lifestyle change to physical activity was also not statistically significant (*p=*0.34) [35]. The progression in long-term care grade indicating a risk of care dependency of older adults was not statistically significant after the multi-component care approach [13]. The pooled effect of the two studies [13, 35] on reducing cardiovascular-related health risks and care dependency was not statistically significant (I^2^=0, *p=*0.426, 95% CI [0.90,1.29]) (see Fig. 4).

#### Cognitive health

**Fig. 5:**
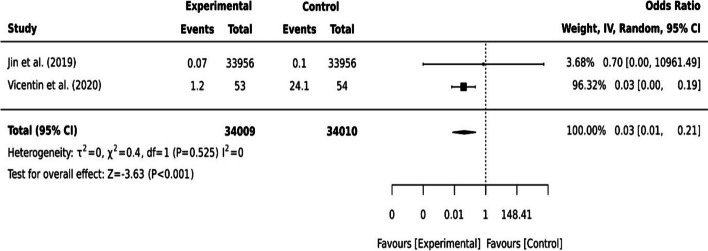
Dichotomous data for two studies on cognition

Digital technology has been utilised to maintain the cognitive health of older adults including slowing cognitive decline and reducing the risk of dementia development [17, 19, 22, 35, 43]. The longitudinal cohort study that observed participants over 8 years using cellphones and desktop devices showed some degree of influence on people’s cognitive functions [19]. The effect of both devices was not statistically different in the 2-year follow-up (*p=*0.30) but different statistically significant in the 4-year follow-up (*p<*0.01) [19]. The study also found different cognition effects between using a cellphone device alone or combined with desktop computer users (*p<*0.01) [19]. In a computer-based digital inclusion with a physical activity program, older adults had an increased score in the Montreal Cognitive Assessment (MoCA) (*p<*0.001) and Mini-Mental State Examination (MMSE) (*p=*0.022) over the 4-month follow-up [43]. However, participants with mild cognitive impairment (Clinical Dementia rating (CDR): 0.5) (*n=*51) showed no statistically significant change (*p=*0.600) [43]. The pooled effect of these two digital interventions [19, 43] on older adults’ cognitive health showed a statistically significant improvement (I^2^=0, *p<*0.001, 95% CI [0.01, 0.21]) (see Fig. 5).

**Fig. 6:**
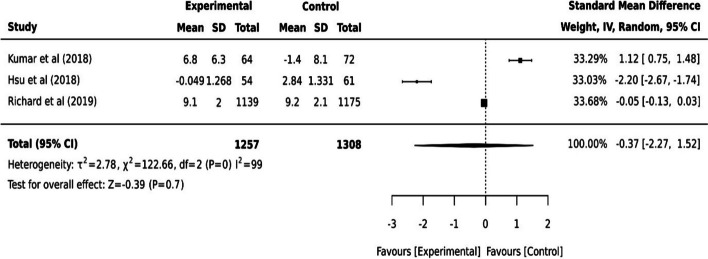
Continuous data for three studies on cognition

Other cognitive health studies have shown various outcomes [17, 22, 35]. A virtual cognitive health program did not show a statistical difference in cognition scores at a 24-week follow-up when measured by Repeatable Battery for the Assessment of Neuropsychological Status scores (RBANS) (*p=*0.15). However, a statistically significant increase in participant cognition was reported at 52 weeks (*p<*0.01) [22]. The secondary effect of the program on older adult’s depression, anxiety and risk of developing dementia also differed statistically significantly from baseline to week 52 (*p<*0.01) [22]. In Richard et al. [35], older adults' dementia risk scores from the Cardiovascular risk factors, Ageing and Incidence of Dementia (CAIDE) showed a statistically significant improvement after the coach-supported internet platform intervention (*p=*0.02). Cognitive health improved in Hsu et al. [17] following digital program intervention, however, it was not statistically significant (*p=*0.132). The variation of the intervention outcomes showed between different timeframes. The pooled effect of these three studies [17, 22, 35] on older adults’ cognitive health was not statistically significant (I^2^=99, *p=*0.7, 95% CI [-2.27, 1.52]) (see Fig. 6).

### Considerations of the digital application to older adults

Thematic analysis was conducted from the qualitative and mixed-method studies and is shown in Table 3. The following sections summarise the emerging themes of digital engagement, communication, independence, human connection, privacy, and cost.

**Table 3:** Thematic analysis

**Themes**	**Code**	**Example quotation**
Digital engagement	Digital literacy/competency	Building on participants’ level of competence through learning and some self-reflection, including knowledge, personal beliefs, and support [33].
		Participants felt that their level of awareness was raised and provided them with encouragement to remain active and meet their goals [27].
		Study products either lacked technological competence or participants preferred communicating through calls instead of text messaging [18].
	Age	Visually impaired participants reported difficulties with the interface and felt those interfaces were probably designed for the younger generation [18].
	Motivation	Ability to adjust the level of exercise based on individual’s condition. Felt that was both important and motivating [33].
		Difficult to find a balance between structure and flexibility [33].
	Person-centred	Participants believed digital tools could positively improve their overall well-being if designed in a patient-centred manner [18].
Communication	Patient and healthcare providers’ communication	Improve and facilitate communication between participants, family caregivers, physicians, and ambulant formal caregivers [18].
		The overall oncology module is useful to improve my communication with my healthcare provider [42].
Independence	Independent living	Reduction of stress/pressure on carers. Increased the level of independence and decrease the level of anxiety for participants [3].
		Being able to remain independent and age in place [18].
Human connection	Human contacts	Fear that technologies might reduce human contacts such as care, empathy, and emotions [18].
		The robot was described as being “too cute” and participants felt offended and described it as “childish”, raising the risk of deception [18].
	Social connection	Moving from own home into a nursing (residential care) home and loss of social connection with the local community. Use Facebook, Skype, text messages, iPad, and smartphone to stay in touch with family members living locally and overseas [4].
		Language decline with the use of English led to a decline in the ability to communicate with staff members. Able to converse with volunteers in their mother tongue through digital technologies – making video calls thus improvement in well-being [4].
Privacy and cost	Privacy/safety concerns	Drawing a line for personal space. Risk of redundant data being collected and repurposed, and a risk of data being misused – stolen, or leaked via a third party [18].
		You have to think about safety. I lose my balance now and then and have to grab hold of a wall or a table when walking by [33].
	Cost concerns	Basic health insurance does not cover reimbursement of digital health technologies, exposing socioeconomic inequalities and low adoption of digital health technologies [18].

#### Digital engagement

Digital engagement in this review refers to the extent to which older adults adhere to or interact with digital intervention. Digital literacy/competency, age, motivation and person-centred were identified to influence digital engagement in older adults [18, 27, 33, 42]. Older age has been viewed as a barrier to the extent of a person’s digital device usage [18, 42]. A generational gap in technology use was found in people aged 80 or older with lower or absent use of digital devices compared to those aged 65 and 79 [18]. Older adults were also less confident in their ability to use the digital tool without any assistance [42]. However, individual preferences and choices of person-centred manner drove positive digital engagement [18]. The flexibility of the programs motivated participants to exercise in their own time [33]. Whereas some participants found it difficult to follow with a lack of clear structure [33].

#### Communication and Independence

Health service communication and the importance of independent living were addressed among older adults [3, 18, 42]. A digital health module that was equipped with a video conferencing feature has enabled older migrants with cancer to communicate with their healthcare providers [42]. Assisted by a smartphone care coordination application, older adults perceived it useful in facilitating communication between patients, family caregivers, and physicians [18]. Additionally, installing a voice control tablet at home for older adults with health conditions enabled them to obtain information and organise personal appointments and medications, positively impacting their independence and reducing stress on carers [3].

#### Human connection

Social isolation and companionship related to human connection were mentioned among older adults [4, 18]. Using video calls or social media platforms, older adults with migration backgrounds could stay connected and maintain their own social and cultural identities [4]. However, older adults expressed fear of reducing human contact with increased technology use [18]. The robotic devices for companionship were found to infantilise general older adults and deceive people living with dementia [18].

#### Privacy and cost

Issues were also raised regarding privacy, safety, and the cost of the technology [6, 18, 33]. Collecting personal information in the digital application could be repurposed, leaked, or accessed by a third party [18]. Health insurance does not cover reimbursement of digital health technologies which may result in socioeconomic inequalities and low adoption of digital health technologies [18]. The cost concern of technology was found to impact participants’ satisfaction with the training program significantly [6].

## Discussion

This review investigated the effectiveness of digital interventions to facilitate healthy ageing and cognitive health and further identified the considerations of its application to older adults. Information, assistive and communication technology were the commonly used types of intervention for older adults. Whilst the study interventions on facilitating healthy ageing were not statistically significant, positive effects were found in the cognitive functions of older adults. The following two sections discuss the effectiveness of the reviewed interventions and considerations of their application to older adults.

### The effectiveness of digital health interventions to facilitate healthy aging and cognitive health in older adults

Digital interventions used in older adults to facilitate healthy ageing were not always effective. The main areas for facilitating healthy ageing from the reviewed studies were summarised into health knowledge, healthy behaviours, physical activities, health risk reduction and care dependency. Health knowledge of the older participants was improved in most digital programs [6, 13, 17, 33, 35]. However, despite the health knowledge was increased among the older participants, the overall health effects on healthy ageing, and health risk reduction were not statistically significant in the meta-analysis [6, 13, 17, 35]. The discrepancy between the individuals’ health knowledge and the health behaviour/implementation is relevant to a study suggesting that health outcomes are not only determined by scientific knowledge improvements but encompass a deeper understanding of one’s perception, choice, and the perceived meaning of a healthy lifestyle [11]. Thus, improving the health knowledge/literacy of individuals does not necessarily result in healthy behaviours and health risk reduction in the older population.

Despite no significant health changes from the improved health knowledge, some older groups were found to particularly benefit from digital interventions, such as carers, immigrants, and people with language barriers. Consistent with the literature, examples were voice-control devices that assist people with chronic diseases and dementia to maintain independent living and reduce carers’ burden [3, 26, 39]. Using social media platforms was found to increase social connection among older immigrants [4]. Health consultation and medical assessment through digitalised care systems created more personalised communication to reduce language barriers and increase social inclusion and health equality for people with non-native-speaking backgrounds [20, 42, 47].

An overall positive effect on participants’ cognitive functions was found from the digital interventions [17, 19, 22, 35, 43]. In particular, using digital devices appeared beneficial to maintain older adults’ cognitive functions in the longer term. Moreover, similar to the literature, computer training programs for physical activity help with cognitive stimulation and maintain older adults’ brain health [5, 43]. Importantly, most of the programs that showed positive effects on cognition were not only approached by brain stimulation alone but included mental and physical activities, nutritional education, social and health consultation etc. This refers to healthy ageing as the foundation for older adults’ cognitive health. As many health risks are known to contribute to cognitive decline and risks for dementia, the risk attribution has led to the cognitive health strategy development being more multi-dimensional [37].

### Considerations of digital application to older adults

As the review identified, health knowledge improvement does not necessarily come with healthy behaviours and risk reduction among older individuals. Therefore, knowledge translation is key to effective intervention. To assist with knowledge translation in the health implementation of older adults, considerations were identified from the reviewed studies. Firstly, the reviewed digital programs were mostly interactive to facilitate self-learning and compose multifaceted health education to suit individual needs and preferences. The person-centred concept seemed to have been integrated into the program designs and had attracted positive user feedback on the accessibility of health information and the flexibility of program engagement. However, digital competency, age, motivation and personal needs influence individuals’ perceptions, choices, and level of personal health engagement; hence, may influence the knowledge translation to the overall health effects on older individuals. Digital competency can be perceived in both ways of learning for individuals to improve digital engagement but also cause disengagement due to a lack of knowledge. Studies found that digital engagement is reduced with increased age [18], a decline in health status is associated with a decrease in technology use [23]. However, this does not mean that engaging in digital activities will result in health effects in older adults but encouraging engagement in digital activities to improve health.

Secondly, older adults are toward a later stage in life with an established lifestyle, social connections, and various physical and psychological health conditions. Therefore, while the interventions attempting to meet individual needs (a person-centred concept) are likely to be adopted by older adults, translating the learned knowledge into everyday life to achieve anticipated health implementation and risk reduction needs further considering the completeness of individuals’ experiences from social, environmental and healthcare systems that often have expanded effects on personal health [21].

Further, the time taken to see the cognition effect from digital devices/interventions seems longer. The average time to see a statistically significant difference in cognitive functions was greater than 4 years [19, 22]. The interventions that were implemented in less than a 2-year timeframe did not have statistically significant effects on individuals’ cognitive functions [19, 43]. This finding was congruent with the fact that both prevention and intervention for cognitive decline in ageing often require a length of time to capture the effects on each individual [34]. This suggests that long-term enhancement and methodological measurement of digital devices are needed for the cognitive health of older adults. Moreover, compared to the non-digital device users, there was a moderately better but not statistically significant cognitive performance of older participants exposed to digital devices and interventions [17], 22, 35. This enhances the daily use of digital devices that may maintain cognitive health for older adults.

Moreover, loss of human contact remains a major concern for older adults, particularly robotic devices replacing conventional human-to-human interaction [18, 40]. The review found that the intervention containing human coaching had more positive outcomes in the studied health areas compared to the interventions without [3, 18, 35]. This suggests that incorporating human factors into digital intervention is needed for older adults [7]. Furthermore, some digital devices require skilled personnel and the relevant health funds are not always available to older adults [18, 42]. This suggests that accessibility and affordability of digital devices require public health initiatives to work in partnership with older adults to strengthen the assessment of individual needs and associated costs.

### Recommendations and implications for practice, policy, and future research

#### Practice

Although individuals’ health knowledge does not necessarily lead to changes in health behaviours, the potential benefits from the overall health knowledge improvements are still acknowledged and should continue being the efforts in future approaches and practices. Indeed, with technologies changing over time, older adults will need to continue learning and practising new skills to bring a more positive impact on own health. Therefore, digital interventions that are designed to facilitate learning and knowledge translation for older adults are inevitably valuable. Moreover, digital engagement emerged as a driving force in knowledge translation and determining whether digital intervention on older adults comes into effect. Digital competency, age, motivation, and meeting individual needs (a person-centred approach) were factors influencing individuals’ digital engagement. Human-to-human interaction (human coaching) was also considered crucial. In essence, meeting individual needs may not sufficiently address the complexity of the health needs of older adults. The expanded meaning of a person-centred concept in older adults (look beyond a person’s health needs from social (i.e., social connection/network impacts), environmental (i.e., health/funding resources) and healthcare systems (i.e., care distribution/equality/communication)) should be pursued. Therefore, future practice is needed to address the factors with a broader person-centred concept to assist older adults with knowledge translation and health implementation.

#### Policy

The concerns of privacy, safety and cost in technology are not new. On the broader level of policy and decision-making, personal data security and a safe digital environment must be protected by government regulations with a standard reviewing process to catch fast-changing technologies. The national standards for digital devices and healthcare systems should be regularly assessed and monitored by policy bodies. In addition, the price value and total cost of technologies need to be supported by healthcare initiatives and funding resources to ensure equality and affordability are maintained for the growing ageing population.

#### Research

Several areas from the reviewed interventions can be further explored and pursued in future research. Firstly, person-centred has been viewed as an important concept when designing digital interventions for older adults. Although the concept seemed to have been integrated into most reviewed studies, the challenge today is how older individuals benefit from the learned knowledge to reduce their health risks. This may mean the investigation into the specific health benefits of digital intervention, for example, by reducing risks in cardiovascular diseases (i.e., hypertension, hyperlipidaemia, stroke), musculoskeletal symptoms (i.e., chronic pain, physical mobility), or mental health conditions (i.e., depression, anxiety). Therefore, future research looking into the specific health benefits would gain a better understanding of the digital phenomenon in older adults.

Secondly, human connection and communication between patients and healthcare providers are important areas of maintaining individual health and independent living. The population ageing and the rising number of older immigrants in most developed countries strongly impact healthcare usage [15, 28]. This implies a need for addressing healthcare inclusion for older immigrants. Thirdly, long-term use of digital devices seems to benefit cognitive health. However, it is unclear whether technology can impact specific cognitive domains. The domain areas of complex attention, executive function, learning and memory, language, perceptual-motor control and social cognition are related to the development of dementia [1]. As the causes of developing dementia are still uncertain to our current knowledge, future research that investigates specific impacts on a cognitive domain from technology applications may provide more understanding about the digital ways of maintaining brain health for older adults.

## Limitations

This review has several limitations in terms of the search strategy and study comparison. As digital health is a broad area, the review limited the search on the topic to only use the keywords search and did not employ mesh terms or expanded words. This has limited the search strategy and may have missed the studies that ought to be included. Cognitive health, the secondary outcome of this review, is also a large topic, therefore this review only included a pragmatic selection of cognitive function measures addressed in the reviewed studies. Moreover, this review primarily focused on healthy older adults and excluded the studies that focused on people with dementia and cognitive impairment. Many intervention studies that focused on dementia and cognitive impairment were also excluded. This narrowed scope has limited the included studies in comparison to dementia risk reduction. Furthermore, the heterogeneous nature of the mixed-method studies limited the comparison of each study intervention.

## Conclusions

The evolution of digital technologies has accelerated its influence on the everyday life of older adults and healthcare. This review evaluated the effectiveness of digital interventions for healthy ageing and cognitive health of older adults through a systematic approach and meta-analysis. Health interventions using digital technology to facilitate healthy ageing of older adults were not always effective. Therefore, knowledge translation into everyday health behaviour to reduce risks is key to effective digital intervention. The overall cognitive functions of older participants were improved by digital interventions; however, it often requires a longer intervention period. Each intervention effect and considerations identified give rise to the areas for future practice, policy, and research. Indeed, technologies will continue to advance, and the perspectives and experiences of older adults on digital approaches to their health may differ from time to time and from generation to generation [36]. Therefore, future work involving digital technology for older adults is necessary to reflect on the intervention effects and considerations identified in this review. Ensuring healthcare innovations can be practically implemented into the everyday life of older adults.

### Supplementary Information

**Additional file 1.**

## Data Availability

The datasets used and/or analysed during the current study are available from the corresponding author on reasonable request.
