# Supplementary material for: Digital interventions for healthy ageing and cognitive health in older adults: a systematic review of mixed method studies and meta-analysis
Source: BMC Geriatr. 2024 Mar 4;24:217. doi: 10.1186/s12877-023-04617-3 (PMC10910826; doi:10.1186/s12877-023-04617-3)
Supplement: Supplementary file 1 — Additional file 1. [file 12877_2023_4617_MOESM1_ESM.docx]

**Supplementary Appendix 1**

**CINAHL**

| Friday, July 22, 2022, 5:23:02 AM |
| --- |

Friday, Sep 9,2022, 2:21PM

| **#** | **Query** | **Limiters/Expanders** | **Last Run Via** | **Results** |
| --- | --- | --- | --- | --- |
| S4 |  | Limiters-2012-2022 and Scholarly Journal |  | 89 |
| S3 | AB digital health AND |  |  | 91 |
|  | AB dementia |  |  |  |
| S2 | AB digital health AND AB older people | Limiters - Published Date: 20120101-20221231 Expanders - Apply equivalent subjects Search modes - Find all my search terms | Interface - EBSCOhost Research Databases Search Screen - Advanced Search Database - CINAHL Complete | 161 |
| S1 | AB digital health AND AB older people | Expanders - Apply equivalent subjects Search modes - Find all my search terms | Interface - EBSCOhost Research Databases Search Screen - Advanced Search Database - CINAHL Complete | 176 |

**ProQuest**

22 July 2022 05:27

8 September 2022 08:01

## Search Strategy

| **Set#** | **Searched for** | **Databases** | **Results** |  |
| --- | --- | --- | --- | --- |
| S2 | ab(digital health) AND ab(older people) AND stype.exact("Scholarly Journals") AND pd(>20120101) | ProQuest Central | 287 | |
| S3 | ab(digital health) AND ab(older people) AND pd(>20120101) | ProQuest Central | 382 | |
| S4 | ab(digital health) AND ab(older people) | ProQuest Central | 504 | |
| S5 | ab(digital health) AND ab(dementia) | ProQuest Central | 229 | |
| S6 | ab(digital health) AND ab(dementia) AND stype exact("Scholarly Journals") | ProQuest Central | 126 | |

**Cochrane**

23 July 2022 11:09

9 September 2022 16:10

| Title/Abstract/Keyword | Digital health | | | | | |
| --- | --- | --- | --- | --- | --- | --- |
| AND | Title/Abstract/Keyword | Older people | | | | |
| Results: 289 | | | | | | |
| Cochrane review: 8 | Cochrane protocol: 0 | Trial: 281 | Editorials: 0 | Special collection: 0 | Clinical Answers: 0 | More |
| Title/Abstract/Keyword | Dementia |  |  |  |  |  |
| Cochrane review: 1 | Cochrane protocol: 0 | Trial: 67 | Editorials: 0 | Special collection: 0 | Clinical Answers: 0 | More |
| Results: 68 |  |  |  |  |  |  |

(Word variations have been searched)

**MEDLINE**

23 July 2022 15:37

9 September 2022 15:03

Ovid MEDLINE(R) ALL <1946 to July 21, 2022>

1 digital health.mp. 5583

2 older people.mp. 36657

3 1 and 2 53

4 dementia.mp 150244

5 1 and 4 14

**Scopus**

23 July 2022 16:10

9 September 2022 15:30

Combine queries

5[Edit](https://www.scopus.com/search/history/edit.uri?shid=4) ( TITLE-ABS-KEY ( digital AND health ) AND TITLE-ABS-KEY (dementia) ( LIMIT-TO ( PUBYEAR , 2022 ) OR LIMIT-TO ( PUBYEAR , 2021 ) OR LIMIT-TO ( PUBYEAR , 2020 ) OR LIMIT-TO ( PUBYEAR , 2019 ) OR LIMIT-TO ( PUBYEAR , 2018 ) OR LIMIT-TO ( PUBYEAR , 2017 ) OR LIMIT-TO ( PUBYEAR , 2016 ) OR LIMIT-TO ( PUBYEAR , 2015 ) OR LIMIT-TO ( PUBYEAR , 2014 ) OR LIMIT-TO ( PUBYEAR , 2013 ) OR LIMIT-TO ( PUBYEAR , 2012 ) ) AND ( LIMIT-TO ( DOCTYPE , "ar" ) ) AND ( LIMIT-TO ( SRCTYPE , "j" ) ) AND ( LIMIT-TO ( LANGUAGE , "english" ) )

[470 results](https://www.scopus.com/search/history/results.uri?origin=searchhistory&shid=4) Set Alert More

4[Edit](https://www.scopus.com/search/history/edit.uri?shid=4) ( TITLE-ABS-KEY ( digital AND health ) AND TITLE-ABS-KEY ( older AND people ) ) AND ( LIMIT-TO ( PUBYEAR , 2022 ) OR LIMIT-TO ( PUBYEAR , 2021 ) OR LIMIT-TO ( PUBYEAR , 2020 ) OR LIMIT-TO ( PUBYEAR , 2019 ) OR LIMIT-TO ( PUBYEAR , 2018 ) OR LIMIT-TO ( PUBYEAR , 2017 ) OR LIMIT-TO ( PUBYEAR , 2016 ) OR LIMIT-TO ( PUBYEAR , 2015 ) OR LIMIT-TO ( PUBYEAR , 2014 ) OR LIMIT-TO ( PUBYEAR , 2013 ) OR LIMIT-TO ( PUBYEAR , 2012 ) ) AND ( LIMIT-TO ( DOCTYPE , "ar" ) ) AND ( LIMIT-TO ( SRCTYPE , "j" ) ) AND ( LIMIT-TO ( LANGUAGE , "english" ) )

[536 results](https://www.scopus.com/search/history/results.uri?origin=searchhistory&shid=4) Set Alert More

3[Edit](https://www.scopus.com/search/history/edit.uri?shid=3) ( TITLE-ABS-KEY ( digital AND health ) AND TITLE-ABS-KEY ( older AND people ) ) AND ( LIMIT-TO ( PUBYEAR , 2022 ) OR LIMIT-TO ( PUBYEAR , 2021 ) OR LIMIT-TO ( PUBYEAR , 2020 ) OR LIMIT-TO ( PUBYEAR , 2019 ) OR LIMIT-TO ( PUBYEAR , 2018 ) OR LIMIT-TO ( PUBYEAR , 2017 ) OR LIMIT-TO ( PUBYEAR , 2016 ) OR LIMIT-TO ( PUBYEAR , 2015 ) OR LIMIT-TO ( PUBYEAR , 2014 ) OR LIMIT-TO ( PUBYEAR , 2013 ) OR LIMIT-TO ( PUBYEAR , 2012 ) ) AND ( LIMIT-TO ( DOCTYPE , "ar" ) ) AND ( LIMIT-TO ( SRCTYPE , "j" ) )

[569 results](https://www.scopus.com/search/history/results.uri?origin=searchhistory&shid=3) Set Alert More

2[Edit](https://www.scopus.com/search/history/edit.uri?shid=2) ( TITLE-ABS-KEY ( digital AND health ) AND TITLE-ABS-KEY ( older AND people ) ) AND ( LIMIT-TO ( PUBYEAR , 2022 ) OR LIMIT-TO ( PUBYEAR , 2021 ) OR LIMIT-TO ( PUBYEAR , 2020 ) OR LIMIT-TO ( PUBYEAR , 2019 ) OR LIMIT-TO ( PUBYEAR , 2018 ) OR LIMIT-TO ( PUBYEAR , 2017 ) OR LIMIT-TO ( PUBYEAR , 2016 ) OR LIMIT-TO ( PUBYEAR , 2015 ) OR LIMIT-TO ( PUBYEAR , 2014 ) OR LIMIT-TO ( PUBYEAR , 2013 ) OR LIMIT-TO ( PUBYEAR , 2012 ) )

[842 results](https://www.scopus.com/search/history/results.uri?origin=searchhistory&shid=2) Set Alert More

1[Edit](https://www.scopus.com/search/history/edit.uri?shid=1) (TITLE-ABS-KEY ( digital AND health ) AND TITLE-ABS-KEY ( older AND people ) )

[905 results](https://www.scopus.com/search/history/results.uri?origin=searchhistory&shid=1) Set Alert More

**PubMed**

24 July 2022 16:49

9 September 2022 16:14

| Search number | Query | Sort By | Filters | Search Details | Results | Time |
| --- | --- | --- | --- | --- | --- | --- |
| 3 | (digital health [Title/Abstract]) AND (dementia) |  | in the last 10 year | ("digital health"[Title/Abstract] AND "older people"[Title/Abstract]) AND "dementia" [Title/Abstract] (y_10[Filter]) | 58 | 16:04 |
| 2 | (digital health [Title/Abstract]) AND (older people [Title/Abstract]) | | in the last 10 years | ("digital health"[Title/Abstract] AND "older people"[Title/Abstract]) AND (y_10[Filter]) | 52 | 2:12:22 |
| 1 | (digital health [Title/Abstract]) AND (older people[Title/Abstract]) | | | "digital health"[Title/Abstract] AND "older people"[Title/Abstract] | 52 | 2:11:46 |
